# Multiplex real-time PCR assay for detection of *Escherichia coli* O157:H7 and screening for non-O157 Shiga toxin-producing *E. coli*

**DOI:** 10.1186/s12866-017-1123-2

**Published:** 2017-11-09

**Authors:** Baoguang Li, Huanli Liu, Weimin Wang

**Affiliations:** 1 Division of Molecular Biology, Center for Food Safety and Applied Nutrition, Food and Drug Administration, Laurel, MD 20708 USA; 2 Branch of Microbiology, Office of Regulatory Affairs, Food and Drug Administration, Jefferson, AR 72079 USA

**Keywords:** Multiplex real-time PCR, *Escherichia coli* O157:H7, Shiga toxins (*stx*1, *stx*2), Shiga toxin-producing *E. col* (STEC), non-O157, Limit of detection (LOD), Pathogen detection, Sensitivity

## Abstract

**Background:**

Shiga toxin-producing *Escherichia coli* (STEC), including *E. coli* O157:H7, are responsible for numerous foodborne outbreaks annually worldwide. *E. coli* O157:H7, as well as pathogenic non-O157:H7 STECs, can cause life-threating complications, such as bloody diarrhea (hemolytic colitis) and hemolytic-uremic syndrome (HUS). Previously, we developed a real-time PCR assay to detect *E. coli* O157:H7 in foods by targeting a unique putative fimbriae protein *Z3276*. To extend the detection spectrum of the assay, we report a multiplex real-time PCR assay to specifically detect *E. coli* O157:H7 and screen for non-O157 STEC by targeting *Z3276* and Shiga toxin genes (*stx*1 and *stx*2). Also, an internal amplification control (IAC) was incorporated into the assay to monitor the amplification efficiency.

**Methods:**

The multiplex real-time PCR assay was developed using the Life Technology ABI 7500 System platform and the standard chemistry. The optimal amplification mixture of the assay contains 12.5 μl of 2 × Universal Master Mix (Life Technology), 200 nM forward and reverse primers, appropriate concentrations of four probes [(*Z3276* (80 nM), stx1 (80 nM), stx2 (20 nM), and IAC (40 nM)], 2 μl of template DNA, and water (to make up to 25 μl in total volume). The amplification conditions of the assay were set as follows: activation of TaqMan at 95 °C for 10 min, then 40 cycles of denaturation at 95 °C for 10 s and annealing/extension at 60 °C for 60 s.

**Results:**

The multiplex assay was optimized for amplification conditions. The limit of detection (LOD) for the multiplex assay was determined to be 200 fg of bacterial DNA, which is equivalent to 40 CFU per reaction which is similar to the LOD generated in single targeted PCRs. Inclusivity and exclusivity determinants were performed with 196 bacterial strains. All *E. coli* O157:H7 (n = 135) were detected as positive and all STEC strains (n = 33) were positive for *stx*1, or *stx*2, or *stx*1 and *stx*2 (Table 1). No cross reactivity was detected with *Salmonella enterica*, *Shigella* strains, or any other pathogenic strains tested.

**Conclusions:**

A multiplex real-time PCR assay that can rapidly and simultaneously detect *E. coli* O157:H7 and screen for non-O157 STEC strains has been developed and assessed for efficacy. The inclusivity and exclusivity tests demonstrated high sensitivity and specificity of the multiplex real-time PCR assay. In addition, this multiplex assay was shown to be effective for the detection of *E. coli* O157:H7 from two common food matrices, beef and spinach, and may be applied for detection of *E. coli* O157:H7 and screening for non-O157 STEC strains from other food matrices as well.

## Background

Shiga toxin-producing *Escherichia coli* (STEC), including *E. coli* O157:H7, is a heterogeneous group of enteric pathogens responsible for numerous sporadic infections and large outbreaks annually worldwide [1]. Besides *E. coli* O157:H7, non-O157 STEC strains are important foodborne pathogens estimated to cause over 112,752 cases illnesses each year in the United States [2]. Shiga toxin (Stx) production, especially Stx2, has been implicated as an important factor in causing severe disease and hemolytic uremic syndrome (HUS) [3–5].

*E. coli* serotype O157:H7 was first recognized as a causative agent of food contamination outbreaks in 1982 in Oregon and Michigan [6]. Since then, numerous *E. coli* O157:H7 outbreaks have been reported worldwide [7]. In the United States alone during 2003 - 2012, 390 outbreaks of *E. coli* O157:H7 infections were documented, which resulted in 4,928 illnesses, 1,272 hospitalizations, and 33 deaths [8]. The typical symptoms caused by *E. coli* O157:H7 include abdominal pain, watery diarrhea and potential progression to bloody diarrhea (hemorrhagic colitis) [9]. The pathological features of hemorrhagic colitis are attributed to the production of Shiga like cytotoxins (Stx1 and Stx2), which consists of a 32-kDa A subunit and five identical 7.7 kDa B subunits. These toxins can bind to receptors located on membranes of eukaryotic cell and cause tissue damage resulting in pathological outcomes [4, 5, 10, 11]. Non-O157 STEC strains are foodborne pathogens and have been responsible for sporadic cases of infections and outbreaks. Although O157:H7 has garnered more attention, primarily based on historical record, recent attention has recognized the significance of non-O157 STEC strains as a pathogen [9, 12–20]. Considerable attention is now drawn to non-O157 STEC strains particularly after the occurrence of a severe foodborne outbreak happened in 2011 in Germany caused by consumption of sprouts contaminated by STEC O104:H4 [21].

The clinical significance and economic burden associated with outbreaks caused by *E. coli* O157:H7 and non-O157 STEC have led to development of a variety of detection methods. These include the application of conventional bacteriological methods using selective media or chromogenic agar, which usually take several days to complete [1, 9, 22], and molecular-based assays such as PCR-based methods [23–25], microarray [25–27], and whole genomic sequencing (WGS) [25, 28, 29]. Of these molecular methods, real-time PCR is a commonly used method [9]. More importantly, real-time PCR enables detection to be coincided with the amplification process by the introduction of fluorogenic probes [23], and multiplex real-time PCR allows multiple genes to be simultaneously amplified either from one template [9] or multiple templates by using different primer pairs [24].

Multiplex real-time PCR has gained more acceptance and use due to its ability to its differentiation potential and reliability [11, 30]. Various target genes have been used in PCR detection scheme for *E.coli* O157:H7, including the Shiga toxin genes (*stx*1 and *stx*2) [9, 12, 13], *eae* [31, 32], *fimA* [33], *rfbE* [34], *uidA* [24, 35], and *Z3276* [23, 36, 37]. Of the target genes, *uidA* is most commonly used. Specificity with this gene is based on a highly conserved point mutation at position 93 of the *β*-glucuronidase gene [38, 39]. However, when a *uidA*-based commercial kit was used for identification of a large number of *E. coli* O157:H7 isolates (n = 391), numerus strains (n = 21) did not generate an amplified product. This prompted us to search for a more specific and reliable gene target for detection of *E. coli* O157:H7 [23]. As a result, a real-time PCR-based on *Z3276* gene, a putative unique fimbriae gene in *E. coli* O157:H7 [40], was developed. All the 391 isolates, including the 21 strains that were “negative” by the *uidA*-based commercial kit, that were tested were positively identified [23]. The primary focus of our previous study was to search for a unique genetic marker and the development of a real-time PCR assay for the detection of *E.coli* O157:H7, and the Shiga toxin genes were not included in that assay [23]. Since the annual number of episodes of domestically acquired foodborne illnesses caused by non-O157 STEC (112,752) is almost doubled that of *E. coli* O157:H7 (63,153) in the United States [2], the inclusion of the *stx* genes can be used as the primary characteristic for STEC detection within the heterogeneous STEC group [41]. Thus, in the present study, we incorporated in a multiplex real-time PCR assay the *Z3276*, *stx*1, and *stx2* genes, as well as an internal amplification control to for detection of *Escherichia coli* O157:H7 and screening for non-O157 STEC. In addition, the assay was assessed with two common food matrices, beef and spinach, for specific detection of *E. coli* O157:H7 and non-O157 STEC strains.

## Methods

### Bacterial strains and growth conditions

All the *E. coli* O157:H7 and non-O157 STEC strains used in this study are listed in Table 1. EDL933 (ATCC 43985) was used as the *E. coli* O157:H7 reference strain. Strains of *E. coli* O157:H7 (n = 135) and non-O157 STEC (n = 33) were used for inclusivity determination. *Salmonella enterica* strains, *Shigella* strains and other pathogenic strains were used for the exclusivity test (Table 2). These strains are all from the strain collections of Division of Molecular Biology, Food and Drug Administration (FDA).

**Table 1:** Results of *E. coli* O157:H7 and non-O157 STEC strains detected by the multiplex real-time PCR assay

Strain	Serotype	Source	Target gene		
			*Z3276*	*stx*1	*stx*2
EC1275	O157:H7	CDC EDL933, hamburger meat	+	+	+
EC1225	O157:H7	WA, 1993	+	+	+
EC1759	O157:H7	USA (MI), 2003	+	+	+
EC1429	O157:H7	Denmark, 1987	+	-	+
EC4420	O157:H7	NY, 12/2006	+	-	+
EC4421	O157:H7	White Onions, NY, 12/2006	+	+	+
EC4422	O157:H7	White Onions, NY, 12/2006	+	+	+
EC4504	O157:H7	MN, 12/2006	+	+	+
EC1428	O157:H7	Argentina, 1977	+	-	+
EC1738	O157:H7	Food isolate	+	-	+
EC1530	O157:H7	Thailand, 1994	+	-	+
EC4440	O157:H7	CDC	+	+	+
EC4163	O157:H7	USA (IL)	+	-	+
EC4438	O157:H7	CDC	+	-	+
EC4431	O157:H7	NY, 12/2006	+	-	+
EC4452	O157:H7	USA (NJ), 2006	+	-	+
EC1709	O157:H7	USA (MI), 2002	+	-	+
EC1760	O157:H7	USA (MI), 2004	+	-	+
EC1431	O157:H7	Japan, 1987	+	-	+
EC4115	O157:H7	Sep-06	+	-	+
EC1734	O157:H7	MA, 2009	+	-	+
EC4302	O157:H7	NY, 12/2006	+	+	+
EC4301	O157:H7	NY, 12/2006	+	+	+
EC4429	O157:H7	NY, 12/2006	+	-	+
EC4428	O157:H7	NY, 12/2006	+	-	+
EC4437	O157:H7	NJ, 12/2006	+	-	+
EC4436	O157:H7	NJ, 12/2006	+	-	+
EC4434	O157:H7	NY, 12/2006	+	-	+
EC4433	O157:H7	NY, 12/2006	+	-	+
EC4432	O157:H7	NY, 12/2006	+	-	+
EC4449	O157:H7	NJ, 12/2006	+	-	+
EC4448	O157:H7	WI, 12/2006	+	-	+
EC4447	O157:H7	CA, 12/2006	+	+	+
EC4201	O157:H7	USA (CA), 2006	+	-	+
EC4456	O157:H7	USA (NJ),2006	+	-	+
EC4461	O157:H7	USA (NJ),2006	+	-	+
EC4502	O157:H7	USA (NJ),2006	+	+	+
EC4503	O157:H7	USA (NJ),2006	+	+	+
EC4505	O157:H7	USA (NJ),2006	+	+	+
EC4511	O157:H7	USA (NJ),2006	+	+	+
EC4200	O157:H7	USA (CA), 2006	+	-	+
EC1267	O157:H7	USA, (NH), 1991	+	+	+
EC1268	O157:H7	USA (MT), 1991	+	+	+
EC4194	O157:H7	USA (CA), 2006	+	-	+
EC1590	O157:H7	USA (WA)	+	+	+
EC4458	O157:H7	USA (NJ),2006	+	-	+
EC4197	O157:H7	USA (CA), 2006	+	-	+
EC1231	O157:H7	USA (WA), 1995	+	+	+
EC4470	O157:H7	USA (NJ),2006	+	-	+
EC4471	O157:H7	USA (NJ),2006	+	-	+
EC1244	O157:H7	USA (GA), 1992	+	+	+
EC4199	O157:H7	USA (CA), 2006	+	-	+
EC1260	O157:H7	USA (GA),1993	+	-	+
EC4167	O157:H7	USA (WI), 2006	+	-	+
EC4168	O157:H7	USA (WI), 2006	+	-	+
EC4193	O157:H7	USA (OH), 2006	+	-	+
EC1265	O157:H7	USA (CA), 1993	+	+	+
EC4184	O157:H7	USA (IL), 2006	+	-	+
EC4204	O157:H7	USA (CA), 2006	+	-	+
EC4171	O157:H7	USA (WI), 2006	+	-	+
EC4205	O157:H7	USA (CA), 2006	+	-	+
EC4206	O157:H7	USA (CA), 2006	+	-	+
EC1593	O157:H7	USA (MI), 2003	+	+	+
EC1239	O157:H7	USA (NE), 1993	+	-	+
EC4174	O157:H7	USA (IL), 2006	+	-	+
EC4187	O157:H7	USA (CT), 2006	+	-	+
EC4188	O157:H7	USA (CT), 2006	+	-	+
EC4191	O157:H7	Spinach, USA (IL), 2006	+	-	+
EC1276	O157:H7	Japan, 1996	+	+	+
EC4501	O157:H7	MN, 12/2006	+	+	+
EC558	O157:H7	Patient raw milk	+	+	+
EC867	O157:H7	USDA-FSIS-380-94	+	+	+
EC874	O157:H7	Apple cider	+	+	+
EC4162	O157:H7	Feces (New Jersey)	+	-	+
EC506	O157:H7	Feces	+	-	+
EC507	O157:H7	Feces	+	+	+
EC4443	O157:H7	CDC	+	+	+
EC4442	O157:H7	CDC	+	+	+
EC4441	O157:H7	CDC	+	+	+
EC4451	O157:H7	NJ, 12/2006	+	-	+
EC4445	O157:H7	NJ, 12/2006	+	+	+
EC4446	O157:H7	NJ, 12/2006	+	+	+
EC1601	O157:H7	USA (MI), 2002	+	+	+
EC1727	O157:H7	MI, 2002	+	+	+
EC1426	O157:H7	Canada, 1988	+	-	+
EC4419	O157:H7	NY, 12/2006	+	-	+
EC4418	O157:H7	NY, 12/2006	+	-	+
EC4417	O157:H7	NY, 12/2006	+	-	+
EC4416	O157:H7	NY, 12/2006	+	-	+
EC4423	O157:H7	NY, 12/2006	+	-	+
EC4424	O157:H7	NY, 12/2006	+	-	+
EC4427	O157:H7	NY, 12/2006	+	-	+
EC4426	O157:H7	NY, 12/2006	+	-	+
EC4435	O157:H7	NY, 12/2006	+	-	+
EC4425	O157:H7	NY, 12/2006	+	-	+
EC4164	O157:H7	USA (WI), 2006	+	-	+
EC4439	O157:H7	MS, 12/2006	+	-	+
EC4450	O157:H7	NJ, 12/2006	+	-	+
EC1217	O157:H7	2003	+	+	+
EC1577	O157:H7	USA (WA), 1995	+	+	+
EC4201	O157:H7	USA (CA), 2006	+	-	+
EC4207	O157:H7	USA (IL), 2006	+	-	+
EC4208	O157:H7	Spinach, USA (IL), 2006	+	-	+
EC1245	O157:H7	USA (GA), 1995	+	+	+
EC4506	O157:H7	USA (MN), 2006	+	+	+
EC4507	O157:H7	USA (MN), 2006	+	+	+
EC4508	O157:H7	USA (MN), 2006	+	+	+
EC4509	O157:H7	USA (MN), 2006	+	+	+
EC4510	O157:H7	USA (MN), 2006	+	+	+
EC1590	O157:H7	USA (WA)	+	+	+
EC1597	O157:H7	USA (CT), 1996	+	+	+
EC1225	O157:H7	USA (WA), 1993	+	+	+
EC1236	O157:H7	Food, USA (CA), 1993	+	+	+
EC1240	O157:H7	USA (OH), 1993	+	+	+
EC1241	O157:H7	Food, USA (OR), 1995	+	+	+
EC4472	O157:H7	USA, (NJ), 2006	+	-	+
EC1242	O157:H7	USA (GA), 1992	+	-	+
EC1243	O157:H7	USA (GA), 1992	+	+	+
EC4463	O157:H7	USA (NJ), 2006	+	-	+
EC4165	O157:H7	USA (WI), 2006	+	-	+
EC4166	O157:H7	USA (WI), 2006	+	-	+
EC4195	O157:H7	Spinach, USA (OH), 2006	+	-	+
EC4192	O157:H7	USA (CA), 2006	+	-	+
EC4182	O157:H7	USA (IL), 2006	+	-	+
EC4469	O157:H7	USA (NJ), 2006	+	-	+
EC4465	O157:H7	USA (NJ), 2006	+	-	+
EC4170	O157:H7	USA (WI), 2006	+	-	+
EC4183	O157:H7	USA (IL), 2006	+	-	+
EC4186	O157:H7	USA (IL), 2006	+	-	+
Ec4169	O157:H7	USA (WI), 2006	+	-	+
EC4189	O157:H7	USA (CT), 2006	+	-	+
EC4175	O157:H7	USA (IL), 2006	+	-	+
EC4176	O157:H7	USA (IL), 2006	+	-	+
EC4444	O157:H7	USA, NJ, 2006	+	+	+
EC4173	O157:H7	USA (IL), 2006	+	-	+
EC1892	O104:H4	STEC, Republic of Georgia, 2011	-	-	+
EC1893	O104:H4	STEC, Republic of Georgia, 2011	-	-	+
EC1891	O104:H4	STEC, Germany, 2011	-	-	+
EC1894	O104:H4	STEC, Germany, 2011	-	-	+
EC1769	O26	STEC	-	+	-
EC1770	O26	STEC	-	+	+
EC1771	O26	STEC	-	+	-
EC1773	O26:H11	STEC	-	+	-
EC1775	O26:H11	STEC	-	+	-
EC1768	O26:H2	STEC	-	+	-
EC1786	O111:NM	STEC	-	+	+
EC1787	O111:H8	STEC	-	+	+
EC1788	O111:NM	STEC	-	+	-
EC1791	O145:H25	STEC	-	-	+
EC1794	O145:NM	STEC	-	+	+
EC1801	O103:H2	STEC	-	+	-
EC1802	O103:H25	STEC	-	+	-
EC1803	O103:H11	STEC	-	+	+
EC1806	O121:H19	STEC	-	-	+
EC1807	O121:H19	STEC	-	-	+
EC1808	O121:H19	STEC	-	+	+
EC331	O26	STEC	-	+	+
EC400	O26:H11	STEC	-	+	+
EC521	O26:H11	STEC	-	+	+
EC540	O26:H-	STEC	-	+	+
EC550	O26:H-	STEC	-	+	+
EC1232	O55:H7	STEC	-	+	+
EC1235	O55:H7	STEC	-	+	+
EC1668	O111:H8	STEC	-	+	-
EC1669	O118:H16	STEC	-	-	+
EC1631	O111:H8	STEC	-	+	-
EC1655	O111:H8	STEC	-	+	-
K12		Negative *E. coli* strain control	-	-	-
NTC			-	-	-

**Table 2:** Detection results of the exclusivity test with different bacterial strains by the multiplex real-time PCR assay

				Target gene		
Genus	Species	Pathotype	Strain name/Serotype	Z*3276*	*stx*1	*stx*2
*Escherichia*	*coli*	EHEC	EDL933/O157:H7	+	+	+
*Escherichia*	*coli*	ETEC	EC1775/O26:H11	**_**	+	**_**
*Escherichia*	*coli*	STEC	EC1803/O103:H11	**_**	+	+
*Escherichia*	*coli*	STEC	EC1807/O121:H19	**_**	**_**	+
*Escherichia*	*coli*	ETEC	EC1801	**_**	+	**_**
*Escherichia*	*coli*	EPEC	EC1501	**_**	**_**	**_**
*Escherichia*	*coli*	EIEC	EC1513	**_**	**_**	**_**
*Escherichia*	*coli*	EDC	DEC5A	**_**	**_**	**_**
*Escherichia*	*coli*		K12/MG1655	**_**	**_**	**_**
				**_**	**_**	**_**
*Salmonella*	*enterica*		SL192/Typhi	**_**	**_**	**_**
*Salmonella*	*enterica*		SL317/Newport	**_**	**_**	**_**
*Salmonella*	*enterica*		SL535/Typhimurim	**_**	**_**	**_**
				**_**	**_**	**_**
*Shigella*	*sonnei*		SH20145	**_**	**_**	**_**
*Shigella*	*dysenteriae*		SH20152	**_**	**_**	**_**
*Shigella*	*flexneri*		SH20155	**_**	**_**	**_**
*Shigella*	*boydii*		SH20140	**_**	**_**	**_**
				**_**	**_**	**_**
*Staphylococcus*	*aureus*		ATCC25923	**_**	**_**	**_**
*Staphylococcus*	*epidermidis*		ATCC12228	**_**	**_**	**_**
*Staphylococcus*	*pyogenes*		ATCC19615	**_**	**_**	**_**
				**_**	**_**	**_**
*Vibrio*	*alginolytica*		ATCC17749	**_**	**_**	**_**
*Vibrio*	*parahemolyticus*		ATCC17802	**_**	**_**	**_**
*Vibrio*	*vulasfians*		ATCC27562	**_**	**_**	**_**
				**_**	**_**	**_**
*Enterobacter*	*cloacae*		ATTCC23355	**_**	**_**	**_**
*Enterobacter*	cloacae		ATCC13047	**_**	**_**	**_**
*Enterobacter*	*cloacae*		ATCC13048	**_**	**_**	**_**
				**_**	**_**	**_**
*Citrobacter*	*freundii*		ATCC8090	**_**	**_**	**_**
				**_**	**_**	**_**
*Klebsiella*	*pneumoniae*		ATCC13883	**_**	**_**	**_**
				**_**	**_**	**_**
*Pseudomonas*	*aeruginosa*		ATCC27853	**_**	**_**	**_**

### Bacterial DNA preparation

Bacteria were grown at 37 °C in Luria-Bertani (LB) broth with agitation at 180 rpm, or on LB agar placed in a gravity convection incubator. Bacterial growth was measured by monitoring the turbidity at 600 nm (OD_600_) using a DU530 spectrophotometer (Beckman, CA). To enumerate bacterial cells, cultures were diluted serially in 10-fold increments with medium and plated on LB agar plates at 37 °C overnight. DNA preparation from bacterial cultures was made with a Puregene cell and tissue kit (Gentra, Minneapolis, MN) as described previously [23]. Briefly, cell pellets from 1 ml of overnight culture were suspended in 3 ml of cell lysis solution and heated to 80 °C for 5 min, followed by addition of 15 μl of RNase A and incubation at 37 °C for 60 min. To remove protein and cell debris, the cell lysate was further mixed with 1 ml of protein precipitation solution, vortexed and centrifuged at 3000 × g. DNA in the supernatant was precipitated by the addition of 2-propanol, centrifuged as above, washed with 70% ethanol, and dissolved in 500 μl of rehydration solution. The concentration of DNA extraction was determined by measuring the optical density (OD_260_) using a NanoDrop spectrophotometer (NanoDrop Technology, Wilmington, DE)

### Primers and probes for the multiplex real-time PCR assay

All the primers, probes, and sequence information are listed in Table 3. The primers and labeled TaqMan probes in this study were designed using Primer Express 3.0 software (Life Technology, Foster City, CA) and synthesized by Life Technology. The primers and probe for *E. coli* O157:H7 specific gene open reading frame (ORF) ORF*Z3276* were described previously [23], the primers and probes for *stx*1, and *stx*2 were designed in this study, and the primers and probe for internal amplification control (IAC) were selected based on the DNA sequence of plasmid pUC19 as previously reported [42]. The IAC was incorporated into the multiplex real-time PCR assay to ensure the amplification is free of inhibitory factors from examined food samples.

**Table 3:** Primers and probes used in the multiplex real-time PCR assay

Target gene	Primer/Probe	Sequence (5' -- 3')	Amplicon	Reference
			length (bp)	
Z*3276*	Z3276 forward	TATTCCGCGATGCTTGTTTTT	130	Li and Chen. 2012
	Z3276 reverse	ATTATCTCACCAGCAAACTGGCGG		
	Z3276 probe	FAM-CCCGCAAATCTTTCCMGBNFQ		
*stx*1	stx1 forward	GGATTTCGTACAACACTGGATGAT	67	This study
	stx1 reverse	ATCCACATCTTCAGCAGTCATTACA		
	stx1 probe	TAMRA-CAGTGGGCGTTCTTMGBNFQ		
*stx*2	stx2 forward	GGGCAGTTATTTTGCTGTGGAT	59	This study
	stx2 reverse	GGTCAAAACGCGCCTGAT		
	stx2 probe	JOE-ACGAGGGCTTGATGTCMGBNFQ		
IAC	IAC forward	CAGGATTAGCAGAGCGAGGTATG	65	Fricker et al. 2007
	IAC reverse	CGTAGTTAGGCCACCACTTCAAG		
	IAC probe	CY5-AGGCGGTGCTACAGAG-MGBNFQ		

### Development of the multiplex real-time PCR assay

The multiplex real-time PCR assay was developed using the Life Technology ABI 7500 System platform and the standard chemistry. The concentrations of primers and probes for each target gene were adjusted to achieve optimal amplification condition. The reaction mixture contains 12.5 μl of 2 × Universal Master Mix (Life Technology), 200 nM forward and reverse primers, appropriate concentrations of four probes [(*Z3276* (80 nM), *stx*1 (80 nM), *stx*2 (20 nM), and IAC (40 nM)] and 2 μl of template DNA. Water was added to make a final reaction volume of 25 μl. The amplification conditions for the multiplex assay were set as follows: activation of TaqMan at 95 °C for 10 min, then 40 cycles of denaturation at 95 °C for 10 s and annealing/extension at 60 °C for 60 s.

To compare the efficiency of the multiplex assay with simplex assay, each of the target genes *Z3276*, *stx*1*,* and *stx*2 was amplified by three individual simplex assays. For the simplex assays, three individual reaction mixtures each contains 12.5 μl of 2 × Universal Master Mix (Life Technology), corresponding forward and reverse primers (200 nM) and probe (100 nM). An equal amount of template DNA (2 μl) was used for the simplex assays, and water was added to make a final reaction volume of 25 μl. The amplification conditions for simplex assays were the same as the multiplex assay.

### Sensitivity test and the limit of detection (LOD) of the multiplex qPCR assay

To determine the sensitivity of the multiplex real-time PCR**,** standard curves of *Z3276*, *stx*1, and *stx*2 in the multiplex real-time PCR were generated. A serial 10-fold dilution from 10 ng to 10 fg/μl of genomic DNA of *E. coli* O157:H7 strain (EDL933) was prepared and 2.0 μl of each dilution was used as template for PCR amplification. The real-time PCR assay was performed using the conditions described as above. The amplification efficiency of the assay was determined using the formula E = (10^-1/slope^-1)*100 [43, 44].

### Inclusivity and exclusivity tests

The inclusivity test for the multiplex real-time PCR was performed with the optimized concentrations for probes *Z3276*, *stx1* and *stx2* on the genomic DNA of *E. coli* O157:H7 strains (n = 135) and non-O157 STEC strains (n = 21) (Table 1). The exclusivity test was performed on various pathogenic strains including strains of EIEC, EPEC, *Shigella,* and *Salmonella* (n = 27) (Table 2). DNA samples were diluted with nuclease-free water to concentration of 50 pg/μl and 2 μl of DNA dilute was used for amplification of target genes. Furthermore, 2 μl of nuclease free water was used to substitute DNA in no template control in the triplex real-time PCR.

### Application of the multiplex real-time PCR assay to detect *E. coli* O157:H7 from spiked spinach and beef

Fresh spinach and beef were purchased from a local retail source and used as food matrices to assess the multiplex real-time PCR assay. These samples were first confirmed to be free of *E. coli* O157:H7 and non-O157 STEC by standard FDA BAM method [45], and subsequently used for the spiking experiments. One for beef (set 1) spinach spiking (set 2). Each set contained six replicates (25 g of beef or spinach), and were inoculated with 80 and 800 CFU/g O157:H7 (EDL933) cells, respectively. Each sample was mixed with 225 ml of LB medium and homogenized for 2 min using a stomacher (Seward, England). The samples were incubated at 37 °C with shaking at 180 rpm for 24 h.

Two ml of the enriched culture was sampled at 0, 4, 8, 12 and 24 h. At these times, the samples were centrifuged at 600 × g for 1 min to remove fat tissues (for beef) or leaf (for spinach) from the samples. The supernatants were transferred to 2-ml microtubes and centrifuged again at 3000 × g for 5 min to collect bacterial cells. The cell pellets were used for DNA extraction with PreMan Ultra Sample Preparation Reagent (Life Technologies), following the instruction of the manufacturer. Two μl of the DNA extraction was used in the multiplex real-time PCR, and roughly 200 copies of plasmid pUC19 DNA (Promega, Madison, MI) was added as template for IAC.

## Results

### Designation and optimization of multiplex real-time PCR

*Z3276* is a unique target gene that was used as the basis for a multiplex real-time PCR assay for the detection of *E. coli* O157:H7. In addition to the *Z3276* marker, three additional targets *stx*1*, stx*2*,* and IAC, were optimized for amplification and detection (data not shown). In order to minimize the interference among probes during the amplification process, the concentration of each probe was titrated and tested with a fixed amount of DNA (1 ng/reaction). The optimal concentrations for probes *Z3276*, *stx*1, *stx*2, IAC were determined as 80 nM, 80 nM, 20 nM, and 40 nM, respectively.

**Fig. 1:**
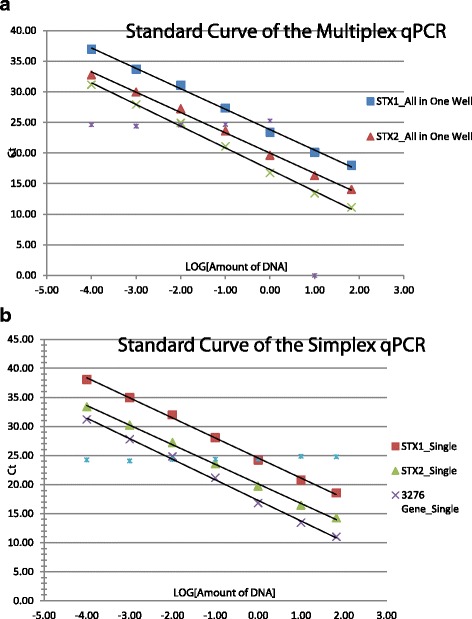
Comparison of the sensitivity of simultaneously detecting the targeting genes *Z3276*, *stx*1, and *stx*2 in *E. coli* O157:H7 by the multiplex real-time PCR assay. The standard curves of *Z3276*, *stx*1, and *stx*2 created by the multiplex real-time PCR assay (**a**); the standard curves of *Z3276*, *stx*1, and *stx*2 generated by three individual simplex real-time PCR assays (**b**). The *C*_*T*_ values represent the average of six replicates from two independent tests ±SD

We further assessed whether the sensitivity of the probes was affected in the multiplex real-time PCR by comparing with those from the corresponding simplex assays. The *C*_*T*_ values for the target genes *Z3276*, *stx*1, and *stx*2 were determined to be 23.86, 22.23, and 21.22, respectively in the multiplex real-time PCR (Fig. 1a), whereas the *C*_*T*_ values for *Z3276*, *stx*1, and *stx*2 were 21.62, 21.38, and 20.63, respectively in simplex real-time PCR (Fig. 1b). These results demonstrated that at a specified amount of target DNA, the sensitivity of the multiplex PCR assay is comparable with that of each respective simplex assay.

### Comparison of sensitivity and specificity of the multiplex and simplex real-time PCR assays

Using a serial 10-fold dilution of genomic DNA from *E. coli* O157:H7 (EDL933) as template, the limit of detection (LOD) in the multiplex real-time PCR was determined to be 200 fg of DNA per reaction with average *C*_*T*_ values of 38.63, 36.21, and 34.04 for *Z3276*, *stx*1, and *stx*2*,* respectively. The value of 200 fg of DNA per reaction is equivalent to 40 CFU of DNA. Three standard curves with slopes of -3.60, -3.49, and -3.36, for the target genes *Z3276*, for *stx*1, and *stx*2*,* respectively, were generated and the amplification efficiencies for the three target genes differed slightly ranging from 90% - 98% (Fig. 1). These data indicated the sensitivity of the multiplex real-time PCR was robust and reliable.

### Inclusivity and exclusivity of the multiplex real-time PCR

The multiplex real-time PCR positively identified all the *E. coli* O157:H7 strains (n = 135) (Table 1). This collection of 135 positively identified *E. coli* O157:H7 strains included the 21 strains that were not amplified in a real-time PCR assay targeting the *uidA* gene [23]. The Shiga toxin profiles (*stx*1 and *stx*2) of these strains were also found to be perfectly matched with those previously determined by conventional PCR and *uidA*-based real-time PCR methods. No cross-reaction was observed from the *E. coli* O157:H7 specific probe *Z3276* on all the non-O157 STEC strains, *Salmonella* strains, *Shigella* strains, and other pathogenic strains; while the *stx* probes positively identified the non-O157 STEC strains with presence of either *stx*1 or *stx*2, or both (Table 1).

### Detection of *E. coli* O157:H7 from spiked food matrices by the multiplex real-time PCR assay

Beef and spinach samples were initially inoculated with 80 CFU/g *E. coli* O157:H7 cells. At 0 h, none of the three detection target genes (*Z3276*, *stx*1, and *stx*2) were detected by multiplex real-time PCR assay, but the IAC was positive. However, after 4-h enrichment, all the three detection target genes (*Z3276*, *stx*1, and *stx*2) from both food matrices were amplified in the multiplex real-time PCR assay (Table 4).

**Table 4:** Detection results of the multiplex real-time PCR on the spiked food samples that inoculated with different concentations of *E. coli* O157:H7 and enriched with different incubation time

Target gene	Food matrice	Incubation time (h)								
	CFU/gram	0	4		8		12		24	
	Beef		C_T_^a^	*±*SD	C_T_	*±*SD	C_T_	*±*SD	C_T_	*±*SD
Z*3276*	80	UD^b^	34.50	0.29	28.43	0.07	27.98	0.10	27.98	0.10
	800	UD	30.51	0.38	24.54	0.10	24.00	0.04	24.00	0.04
*stx*1	80	UD	27.54	0.19	21.73	0.22	20.84	0.25	19.06	0.36
	800	UD	24.45	0.22	18.51	0.14	17.46	0.03	18.48	0.02
*stx*2	80	UD	31.34	0.10	25.47	0.02	25.03	0.04	23.63	0.02
	800	UD	27.93	0.34	21.68	0.03	21.33	0.06	22.52	0.05
IAC	200 copies^c^	28.88	27.72		21.96		21.09		19.59	
	Spinach		C_T_	*±*SD	C_T_	*±*SD	C_T_	*±*SD	C_T_	*±*SD
Z*3276*	80	UD	33.92	0.04	24.32	0.26	22.86	0.15	24.26	0.09
	800	UD	30.91	0.05	23.04	0.11	23.08	0.08	23.12	0.07
*stx*1	80	UD	27.63	0.16	17.32	0.15	15.74	0.13	16.87	0.11
	800	UD	25.04	0.06	16.57	0.12	16.25	0.15	16.37	0.10
*stx*2	80	UD	30.88	0.06	21.21	0.07	20.10	0.06	21.64	0.07
	800	UD	28.19	0.14	20.05	0.15	20.58	0.05	20.74	0.04
IAC	200 copies	24.94	23.82		24.77		23.83		23.69	

^a^Data were shown as average of two independent experiment

^b^UD refers to "Underdetermined", a negative detection result

^c^About 200 copies of pUC19 plasmid DNA was added as template for IAC to each multiplex real-time PCR reaction

## Discussion

PCR technology is widely used for pathogen detection from clinical, food, and environment samples. Real-time PCR methods are used for their enhanced sensitivity and specificity. Several PCR-based methods are available for the detection of *E. coli* O157:H7 and non-O157 STEC (4, 7, 9, 10) by amplifying various target genes [23]. Target genes such as *stx* [9, 12–20], *eae* [31, 32], *fimA* [33], *rfbE* [34] have been used in various assays for detection of for *E. coli* O157:H7, however, most of those genes are not unique genetic markers for this pathogen [23]. This inadequate discriminatory power of those target genes calls for selection of more genetic markers for *E. coli* O157:H7. Consequently, we identified *Z3276* as a unique genetic marker for detection of *E. coli* O157:H7 [23, 36], and confirmed by other scientific groups [37, 46].

Selection of *Z3276* as a unique genetic marker for detection of *E. coli* O157:H7 was the basis for the development of a multiplex PCR assay. Simultaneous detection of multiple genes in a single reaction may increase specificity and reliability for the detection of *E. coli* O157:H7, since the amplification of different target genes can corroborate the final conclusion. More importantly, the inclusion of the Shiga toxin genes enables the assay to detect not only *E. coli* O157:H7, but also screen for non-O157 STEC strains, the latter often underestimated [47–53].

Multiplex real-time PCR can provide better detection efficiency. However, interference among probes and competition among primers for supplies during amplification may compromise the sensitivity and increase the background. Therefore, it is necessary to fine tune the parameters of the multiplex reaction to achieve the optimal conditions for each target gene. In this study, by optimizing the concentration of each probe in the assay (*Z3276*, *stx*1, and *stx*2), we were able to achieve robust sensitivity in the multiplex assay, and positively identified all the *E. coli* O157:H7 strains (n = 135), demonstrating the multiplex assay is compatible to the simplex assays.

Nowadays, WGS has been explored for a more efficient and more comprehensive approach for STEC detection. Although WGS potential with STEC characterization and surveillance is apparent, STEC detection will likely continue to rely on a combination of culture and non-culture methods, the latter including real-time PCR [49]. The multiplex real-time PCR developed in this study not only can detect *E. coli* O157:H7 and its profile of the Shiga toxin genes, but also detect non-O157 STEC strains. The capability for simultaneous detection of the Shiga toxin genes and the differentiation of *E. coli* O157:H7 from non-O157 STEC strains offers several advantages: i) determination of the presence or absence of Shiga toxin genes can be used to verify the detection results of *E. coli* O157:H7, because almost all *E. coli* O157:H7 strains possess *stx*1 and/or *stx*2 gene(s); ii) profile of the Shiga toxin genes of *E. coli* O157:H7 provides genetic markers for differentiating isolates from outbreaks; iii) differentiation of *stx*1 and *stx*2 harboring *E. coli* O157:H7 strains may help health care providers manage HUS patients caused by *E. coli* O157:H7 [49]; and iv) identification of *stx* gene harboring isolates can serve as a useful clue for detection of STEC, and then more comprehensive and sophistical analytical analyses, such as cultural biological tests, toxin detection, serotyping, genotyping, and WGS, can be performed to confirm the final detection result.

Multiplex real-time PCR targeting *uidA*, *stx*1, and *stx*2 genes for detection of *E. coli* O157:H7 and non-O157 STECs has become a routine test for preliminary screening in clinical laboratories as the Centers for Disease Control and Prevention recommended [54]. Although WGS is not yet a routine testing, future prediction would include this technology as a means to track the mobility of pathogenic microbes as the food market has become global. The multiplex real-time PCR developed in this study has been demonstrated to be a reliable, efficient, and sensitive assay, and may serve as a useful method for the detection of *E. coli* O157:H7 and non-O157 STEC in epidemiological surveillance programs as well as in food analytical laboratories. The multiplex real-time PCR assay was successfully tested in this study for the detection of *E. coli* O157:H7 from spiked food matrices, i.e., beef and spinach, and more than likely applicable to other food matrices.

It is worth noting that on the one hand, in the development of the multiplex real-time PCR assay, great efforts were made toward getting high sensitivity and specificity by optimizing the amplification conditions and by minimizing the interferences among probes, primers, and target genes to reduce the false negative rate in detection; on the other hand, in the use of the assay, precaution is needed in interpretation of the positive results from certain strains that free phages might harbor *stx* gene [55–57] or even some *Shigella* strains acquired *stx* genes [58–61].

## Conclusions

A multiplex real-time PCR assay that can rapidly and simultaneously detect *E. coli* O157:H7 and screen for non-O157 STEC strains has been developed and assessed for efficacy. The inclusivity and exclusivity tests demonstrated high sensitivity and specificity of the multiplex real-time PCR assay. In addition, this multiplex assay was shown to be effective for the detection of *E. coli* O157:H7 from two common food matrices, beef and spinach, and may be applied for detection of *E. coli* O157:H7 and screening for non-O157 STEC strains from other food matrices as well.

## Abbreviations

HUS: hemolytic-uremic syndrome
IAC: internal amplification control
ORF: open reading frame
STEC: Shiga toxin-producing *Escherichia coli*
